# Decision Support Systems in Prostate Cancer Treatment: An Overview

**DOI:** 10.1155/2019/4961768

**Published:** 2019-06-06

**Authors:** Y. van Wijk, I. Halilaj, E. van Limbergen, S. Walsh, L. Lutgens, P. Lambin, B. G. L. Vanneste

**Affiliations:** ^1^The D-Lab: Decision Support for Precision Medicine GROW - School for Oncology and Developmental Biology & MCCC, Maastricht University Medical Center, Maastricht, Netherlands; ^2^Department of Radiation Oncology (MAASTRO), GROW - School for Oncology and Developmental Biology, Maastricht University Medical Center, Maastricht, Netherlands

## Abstract

**Background:**

A multifactorial decision support system (mDSS) is a tool designed to improve the clinical decision-making process, while using clinical inputs for an individual patient to generate case-specific advice. The study provides an overview of the literature to analyze current available mDSS focused on prostate cancer (PCa), in order to better understand the availability of decision support tools as well as where the current literature is lacking.

**Methods:**

We performed a MEDLINE literature search in July 2018. We divided the included studies into different sections: diagnostic, which aids in detection or staging of PCa; treatment, supporting the decision between treatment modalities; and patient, which focusses on informing the patient. We manually screened and excluded studies that did not contain an mDSS concerning prostate cancer and study proposals.

**Results:**

Our search resulted in twelve diagnostic mDSS; six treatment mDSS; two patient mDSS; and eight papers that could improve mDSS.

**Conclusions:**

Diagnosis mDSS is well represented in the literature as well as treatment mDSS considering external-beam radiotherapy; however, there is a lack of mDSS for other treatment modalities. The development of patient decision aids is a new field of research, and few successes have been made for PCa patients. These tools can improve personalized medicine but need to overcome a number of difficulties to be successful and require more research.

## 1. Introduction

Worldwide, prostate cancer (PCa) is the second most occurring type of cancer in men and the most commonly diagnosed cancer for men living in developed countries, making it a very relevant topic for cancer research [1].

A variety of treatment options is available to treat PCa, such as external-beam radiotherapy (EBRT) and radical prostatectomy [2–4], which have similar long-term survival outcomes. Other treatments such as brachytherapy [5–8] are gaining popularity, and active surveillance is an increasingly viable option as well [9, 10], due to the slow progression of some kinds of PCa. Retrospective studies comparing different treatment modalities tend to be conflicting and biased. Consensus on the best treatment choices for men with PCa remains absent because prospective trials for different treatments report different toxicities [4, 11, 12]. Due to this, the treatment choice is largely dependent on both patient and physician subjective preferences, rather than knowledge-based decision-making [13]. Additionally, treatment outcome is dependent on a large number of features, including treatment, patient, tumor, clinical, and genetic features [14]. These factors further complicate the integration of evidence-based decision-making into clinical practice due to the limitations of human cognitive capacity, which can only take a relatively small number of factors into account on which to base a decision [15, 16]. In order to meet the aspiration of personalized medicine, the need for multifactorial decision support systems (mDSS) is growing [17–23]. An mDSS is a tool designed to improve the difficult medical decision-making process. It uses multifactorial inputs (treatment, patient, tumor, clinical, genetic, etc.) for a given patient to generate case-specific advice for patients, clinicians, or other medical professionals. Due to the variety of treatment options for PCa, all equally efficacious for outcome, but having different secondary effects, this disease is an interesting subject for the use of mDSS.

In addition to the need for mDSS for treatment selection, similar systems can be used for the diagnosis of PCa, improving early detection as well as reducing overdiagnosis and unnecessary testing. These mDSS can use imaging, clinical, biological, and other parameters to improve detection and risk classification of PCa in a minimally invasive method to maximize individual treatment.

This study provides an overview of the literature to analyze current available mDSS focused on PCa, in order to better understand the availability of mDSS as well as noting where the current literature is lacking. We aim to provide an update for clinicians about recent advances in mDSS for personalized PCa oncology, which may improve clinical decision-making.

## 2. Materials and Methods

### 2.1. Search Strategy

**Figure 1:**
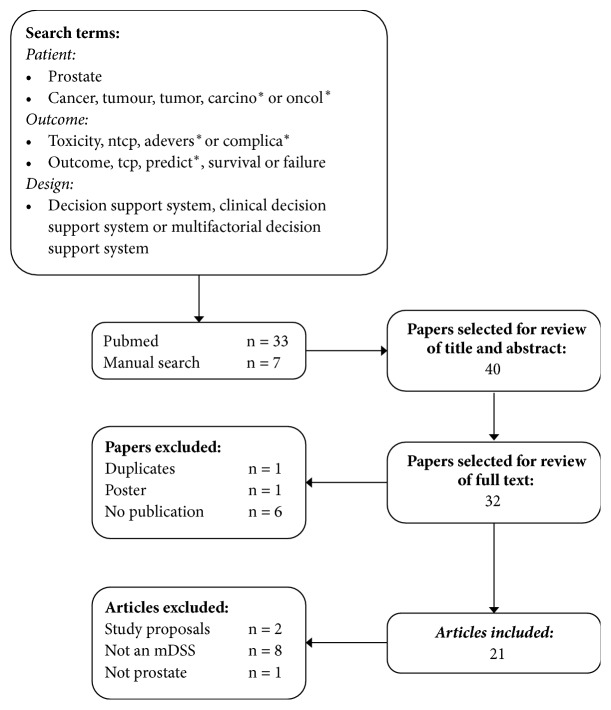
Flowchart of the results of the literature search in PubMed.

In order to identify all mDSS with relation to treatment for PCa, we performed a MEDLINE/PubMed literature search in July 2018, restricted to English. Details of the strategy we used are shown in Figure 1.

### 2.2. Selection Criteria

Prior to reviewing full texts, we manually checked the abstracts and titles to select papers for this study. Duplicates, posters, or abstracts that did not include a published work were excluded. Additionally, we excluded studies which clearly did not include an mDSS for PCa. After full text review, we excluded any papers that described study proposals that did not describe an mDSS in PCa. We selected appropriate studies by manually screening and considering the aforementioned criteria.

### 2.3. Study Characteristics

The studies we included were divided into sections according to the type of mDSS: diagnostic mDSS, which support the staging of PCa or support the decision for more invasive or expensive diagnostic tests; treatment mDSS, which support the decision between treatment modalities or treatment plans; and patient mDSS, which focus on informing the patient.

We described each study using the number of patients, the decision that the system supports, and the system inputs and outcomes. We also commented on the general applicability, the reported performance of the system, and the limitations. In order to assess the reporting quality of the studies, we tested each paper for its compliance to the TRIPOD (Transparent Reporting of studies on prediction models for Individual Prognosis Or Diagnosis) reporting guideline [24]. We reported this in a percentage calculated using the document: “Appendix I: Scoring adherence of prediction model study reports to the TRIPOD reporting guideline”, available on http://www.tripod-statement.org/ (available here).

## 3. Results

### 3.1. Included Studies

#### 3.1.1. mDSS for Diagnosis and Diagnostic Interventions

The studies that contained a diagnostic mDSS are listed in Table 1. Two of the studies we found had the goal of supporting the use of a diagnostic tool: Lee et al. (2010) and Mukai et al. (2013). The application tested by Mukai et al. (2013) was meant to support the use of prostate specific antigen (PSA) tests for patients. The mDSS was a web-based application that would aid in the decision to perform PSA tests for general practitioners (GPs) in Denmark. The study leads to the conclusion that it was possible to grant GPs in Denmark easy access to web-based mDSS by replacing certain words in their medical records by hyperlinks. However it also showed that this mDSS did not change PSA-testing behavior. Since this study neither developed nor validated this mDSS, the compliance to the TRIPOD guidelines could not be tested.

Lee et al. (2010) attempted to support the use of a biopsy by predicting initial biopsy outcomes through three different models: support vector machine (SVM), artificial neural network (ANN), and multiple logistic regression. The study trained each of the models on 600 patients who had undergone transrectal ultrasonography (TRUS)-guided prostate biopsies, tested them on 477, and compared the model performances. The parameters of the models were TRUS findings and clinical parameters, including age and PSA. The area under the receiver operating characteristic (ROC) curve (AUC) for the use of multiple logistic regression analysis, ANN, and the SVM was 0.768, 0.778, and 0.847, respectively, and pairwise comparison of the ROC curves showed that the SVM model had superior performance.

Kim et al. (2011), Sadoughi et al. (2014), Shah et al. (2012), van Leeuwen et al. (2017), and Chang et al. (1999) all aimed to detect, diagnose, or classify PCa using a variety of methods. Kim et al. (2011) performed a study similar to Lee et al. (2010), but with the aim of improving pathological staging, rather than reducing the number of biopsies. Two models were developed, SVM and ANN, for the prediction of advanced PCa and compared based on performance. The models used TRUS-guided biopsy parameters and were tested on 532 patients divided into training and test groups. The SVM model performed significantly better (p=0.02) than the ANN model based on ROC curve, with an AUC of 0.805 while that of the ANN model was 0.719. This study showed that these models could improve objective pathological staging of biopsy-proven PCa patients and could be applied in combination with TRUS-guided biopsies once externally validated.

Another neural network was trained on laboratory results by Sadoughi et al. (2014) who then performed particle swarm optimization. The specific goal of this study was to aid in distinguishing between localized PCa and benign hyperplasia of the prostate. The model was internally validated on 60 patients, and the authors found an accuracy of 98.33%. The description of the methodology was limited, and the reporting conformed only to 33% of the TRIPOD guidelines. The model could potentially improve detection of PCa and possibly reduce the number of biopsies, but external validation is necessary.

Notable were Shah et al. (2012), who also used SVM, but, in contrast to Lee et al. (2011), the model was not trained on biopsy results, but on pathological regions of a magnetic resonance imaging (MRI) scan of postprostatectomy prostates. The aim was not only to diagnose PCa, but to locate it specifically on MRI scans by modeling voxel specific risk analyses. The sensitivity and the specificity of the model with optimized SVM parameters were 90%, and the kappa coefficient was 80%, where the raters were the mDSS and the ground truth histology. The study only included 24 patients, but since the model was trained on specific regions, the training was done on 225 cancer and 264 noncancerous regions. This model could be applied in any hospital with a 3.0 T endorectal multiparametric magnetic resonance imaging (mpMRI) scanner, although it still requires validation.

Van Leeuwen et al. (2017) developed a nomogram, rather than a deep learning algorithm, that included a larger number of parameters to diagnose significant PCa. The nomogram included Prostate Imaging Reporting and Data System (PIRADS), age, PSA, digital rectal examination (DRE), prostate volume, and prior biopsy. The model performed with an AUC of 0.864 on an external validation set, and the paper proposed an optimal strategy to reduce the number of biopsies needed with minimal risk of underdiagnosis. This paper conformed to 97% of the TRIPOD guidelines.

Chang et al. (1999) evaluated the usefulness and the performance of an mDSS, the Prostate Cancer Expert System (PCES), which was validated on 43 patients with confirmed PCa. The PCES system, which utilized PSA, Gleason score, TRUS, and DRE, was used to categorize the patients into localized or advanced PCa, and the same test was applied to four attending physicians and four residents. The results showed that the PCES performed with a higher accuracy than all residents and physicians, though the difference was only higher for two physicians. It also showed that after consultation of the PCES, the staging accuracy of the residents improved to the level of the attending physicians.

A number of prediction tools are currently being applied in the clinic to aid in the further diagnosis of the PCa disease, providing predictions on lymph node (LN) involvement, organ confinement (OC), seminal vesicle (SV) involvement and extracapsular extension, and risk of failure after treatment. The Partin tables [37, 38] are a set of nomograms to predict OC of PCa, initially introduced in 1993 and most recently updated by Tosoian et al. (2017) [36]. Based on these nomograms, Roach derived a set of formulas for the prediction of SV involvement in a paper published by Diaz et al. (1994) [26]. Roach et al. (1994) [25] also derived formulas that predicted LN involvement based on PSA and Gleason score, and Roach et al. (2000) [29] did the same for the risk of failure following radiotherapy (RT) and extracapsular extension in patients with localized PCa.

Tosoian et al. (2017) validated and updated the Partin tables on a cohort of 4459 patients with the goal of predicting the pathological outcome after radical prostatectomy. The performance of the model was tested for binary regression where the AUC was calculated when comparing organ confined (OC) PCa to other pathological outcomes. The model performed best when predicting OC versus LN involvement (AUC = 0.918) and versus seminal vesicle (SV) involvement (AUC = 0.856). The weakest performance was for OC versus extraprostatic extension (AUC = 0.673).

Diaz et al. (1994) split patients into high risk and low risk groups of SV involvement using PSA and Gleason score and tested this on 217 patients. The incidence rate of SV involvement in the low risk group was 7%, while the incidence rate in the high risk group was 37%, resulting in a chi-square of 23.17.

Roach et al. (1994) performed a similar study on 282 patients, but divided the patients into low and high risk groups for LN involvement. This resulted in 6% incidence rate in the low risk group and a 40% incidence rate in the high risk group, resulting in a significant split (p <0.001).

Roach et al. (2000) split 895 patients into low, intermediate, and high risk groups for extracapsular extension. This resulted in an incident rate of 17.8%, 46.7%, and 66.7% in low, intermediate, and high risk groups, respectively, which was a significant split (p <0.01).

D'Amico et al. (1998) suggested a widely accepted risk classification for prostate cancer to help predict biochemical outcome after five years after PCa treatment, stratifying them into low, intermediate, and high risk PCa [27]. This study included 1872 patients who underwent radical prostatectomy, EBRT, or interstitial RT. Cox regression was used to calculate the relative risk between different groups of patients, based on risk level and treatment type. No validation was performed in this paper.

Memorial Sloan Kettering Cancer Center (MSKCC) also has a publically available set of nomograms that is based on data from more than 10,000 patients. They have nomograms available to predict outcome after radical prostatectomy both before and after treatment as well as after PSA elevation (see https://www.mskcc.org/nomograms).

#### 3.1.2. mDSS Supporting Treatment Decisions

The studies that contained treatment mDSS are listed in Table 2. The studies described by Walsh et al. (2018), Smith et al. (2016), and van Wijk et al. (2018) compare different RT treatment plans performed on the same patients and the mDSS selects the best plan with most favorable outcome. This type of personalized mDSS is very suitable for the comparison of RT modalities, as the treatment plans are a predictor for the delivered dose, with consequent treatment outcome. Walsh et al. (2018) used a combination of existing models for tumor control probability (TCP) and normal tissue complication probability (NTCP) to compare different treatment plans with photon and proton RT for 25 patients. The study included extensive corrections for displacements during treatment, which aided in the prediction of delivered dose. As this was a modeling study, no validation was done. However, the concept could be used as a basis for RT plan selection between different modalities and could aid in the optimization of TCP and NTCP. Smith et al. (2016) utilized an advanced Bayesian network to optimize intensity modulated radiotherapy (IMRT) treatment plans based on outcome in terms of progression free survival and toxicity. The models were validated against independent clinical trials for the metastasis free survival and overall survival and resulted in uncertainties of 2.5% and 2%, respectively. This method could potentially be implemented into any IMRT planning system and has the potential to improve the quality of treatment plans, resulting in optimized outcomes.

A device called the implantable rectum spacer (IRS) has been developed to spare the rectum during IMRT by increasing the distance between the anterior rectum wall and the prostate [39, 40]. Van Wijk et al. (2018) made use of image deformation based on a* virtual* IRS [41, 42]: she published models to predict the sparing effect of an IRS before implanting the IRS. The model was tested in a proof of concept study with 16 patients, comparing the mDSS outcome for the* virtual* IRS to the* real* IRS in the patients, and the median discrepancies in outcome were 1.8%. Once validation has taken place, this DSS could be applied to any RT planning system and has the potential to personalize treatment choice.

Two studies that supported the decisions involving follow-up treatment were found. Reed et al. (2014) analyzed the cost-effectiveness (CE) of the use of a nucleic acid detection immunoassay (NADiA) ProsVue™ to support the decision for adjuvant radiotherapy (ART). This model showed that primarily for the intermediate risk patients, NADiA ProsVue had an incremental cost-effectiveness ratio (ICER) lower than $50,000 in 83.6% of the simulations. This analysis in its current state is not applicable outside the USA. Salem et al. (2018) validated a computer-led decision support system aimed at giving additional advice in follow-up treatment strategy. This system uses the clinical profile of the patient in combination with blood test results to propose a follow-up strategy. Validation was done on 200 patients by comparing the computer generated strategy to the advice given by trained urologists. In all cases, the agreement between the experts and the system was better than moderate (kappa >0.6). The paper argued that such a system could significantly reduce costs in follow-up care in the United Kingdom and believed the system can be used by any healthcare worker, regardless of urology background.

Hodges et al. (2012) developed a CE model to analyze the benefit of IMRT with respect to stereotactic body radiotherapy (SBRT). Though this study compared these two treatments, this study carried out analysis on cohort level and not case-specific, as is expected of personalized medicine. The CE analysis was done solely based on probabilistic simulations, thus not taking into account variations in cost, transition rates, or utility values. A sensitivity analysis was performed, revealing that in 66% of the iterations SBRT was cost-effective over IMRT.

Vanneste et al. (2015) constructed a CE model to compare IMRT + IRS with IMRT without IRS. He developed a decision-analytic Markov model to examine the effect of late rectal toxicity and compare the costs and quality-adjusted life years (QALYs). An ICER of €55,880 per QALY was gained. For a ceiling ratio of €80,000, IMRT+S had a 77% probability of being cost-effective.

#### 3.1.3. mDSS Focusing on Providing Patient Information

This section describes the studies that attempted to improve shared decision-making. This type of mDSS fosters patient involvement in therapeutic decisions and emphasizes the provision of information needed to make such decisions (Table 3). Berry et al. (2013) described the testing of a personal patient-profile (P3P) intervention for PCa patients. P3P is a web-based program to help men prepare for shared decision-making about the management of early stage prostate cancer. The study hypothesized that when P3P was used in combination with a consultation with different clinicians to prepare for decision-making, patients were more satisfied with their treatment choice and experienced less regret, but testing did not result in significant improvement. However, this type of system has the capability to take into account patient preferences and priorities, while when only using consultations, these preferences can be misinterpreted by clinicians. The paper suggested similar studies to be performed in the future on larger cohorts.

Nguyen et al. (2009) performed an extensive literature research for predictive outcome models for 15 different treatment options for PCa, including active surveillance, combinations of radical prostatectomy, RT, hormonal therapy, and high intensity focused ultrasound. They attempted to create a comprehensive overview of the different outcome combinations, such as survival, metastasis, and various toxicities. This overview was designed to be comprehensive for patients so that they could use their own priorities and preferences to make an informed decision. Though they concluded that there is a great need for additional models, this paper provided a framework and is a step towards evidence-based personalized medicine. Additionally, this framework could be a useful tool for clinical decision-making by medical personnel when adapted for these users.

### 3.2. Excluded Studies

Two studies, Stacey et al. (2016) [52] and Jayadevappa et al. (2015) [53], were excluded because they contained study proposals. Both these studies will attempt to test patient decision aids. Another study, McRae et al. (2016) [54], was excluded because it was not about prostate cancer.

Eight studies in total were excluded as they did not describe an mDSS, but instead provided tools that could be used in the development of an mDSS. Though these studies can be used to further personalize PCa treatment, these tools cannot be applied in the clinic in their current form. A short overview of these papers is given in Table 4.

Two studies, Daemen et al. (2009) and Beyan et al. (2014), attempted to use genetic information to improve cancer prediction outcomes. Beyan specifically worked to incorporate single nucleotide polymorphisms (SNPs) into the national health information system of Turkey.

Zumsteg et al. (2013) [55], Gnanapragasam et al. (2016) [56], and Epstein et al. (2016) [57] provided new definitions for risk group stratification, increasing upon the current low, intermediate, and high risk groups. They found clear separation in progression free survival in their new risk groups, making these a potential tool for diagnostic mDSS. Gries et al. (2017) [58] provided a method to interpolate the utility values for all combinations of 18 different health states, which could be a valuable tool for mDSS to quantify QALYs. Kuru et al. (2013) [59]showed that a PIRADS score on mpMRI is prognostic for PCa.

Finally Kent et al. (2015) [60] performed a literature search and attempted to create a diagnostic tool which would predict the life expectancy in PCa patients. They concluded that no existing model was suitable for incorporation into an mDSS.

## 4. Discussion

### 4.1. Primary Findings

In response to the increasing number of PCa treatment options, the development of mDSS has become a growing topic of interest to provide an aid in difficult medical decisions. Diagnosis and staging mDSS have also been a growing topic of interest, as well as a number of tools to improve cancer detection, predictions of treatment outcome, and outcome stratification. However, there is still a need for new mDSS in treatment decision aids and validation of diagnostic mDSS. Additionally, the field of patient informed decision-making is still in its infancy, but essential for the growth towards individualized medicine.

For the diagnostic tools, there are a number of viable tools available for the diagnosis of local PCa that have been validated on large cohorts [30, 31, 33, 35, 63]. Additionally, a study has been performed for detection of PCa on the voxel level for MRI images [32]. This type of diagnostic mDSS could additionally assist in treatment planning or treatment selection. One study explores diagnosis of PCa with LN metastasis [64], but this field remains largely uncharted, similar to the computer automated detection of biochemical failure or treatment failure, also explored in only one study [48].

For treatment mDSS, the proportion between the number of treatment options and the decision support tools remains somewhat skewed. Nguyen et al. (2009)[50] suggested 15 different combinations of treatment options, and this did not include the use of rectal displacement devices [65, 66] or proton therapy. The treatment mDSS that were found by the current study primarily involved RT, including treatment plan selection [46], proton compared to photon therapy [49], SBRT compared to IMRT [43], and the use of an implantable rectum spacer in EBRT [47]. Nguyen et al. (2009) attempted to create a general overview of prediction tools to create a clear overview for patients, and they found most prediction tools to be focused on RT and radical prostatectomy. Less prediction tools are available for brachytherapy, which is a very viable treatment option for PCa. One study, Alitto et al. (2017) [67], describes an Umbrella Protocol for the standardized development of new mDSS. This protocol could help in improving the application of new mDSS in clinics.

Development of patient decision aids is challenging, as cultural and language barriers are much more present in this field of research. Nguyen et al. (2009) developed a comprehensive treatment overview for patients, but found that the predictive tools available were limited, leaving an incomplete overview. Berry et al. (2013) [51] hypothesized that patients satisfaction was increased after treatment when they were actively involved in the decision-making process and comorbidities like anxiety, depression, and fatigue were reduced, and they proceeded to test this using the P3P intervention. They have found, however, that this method has not increased self-reported preparation for the intervention.

### 4.2. mDSS Design

The last decade deep learning algorithms have gained popularity in the development of mDSS for the classification of cancers, and the same is true within the field of PCa. Kim et al. (2011) and Lee et al. (2010) tackled a similar problem with both an ANN and an SVM, but the dataset of Kim et al. (2011) was approximately double in size. This increased patient cohort resulted in a higher AUC performance for both models, which confirms that deep learning algorithms rely heavily on large datasets. Also, no external validation was done, so currently these models are not generally applicable. An approach to make these models both more accurate and more applicable for clinical use would be to use distributed learning, where the models are trained on centers all over the world, without the data having to leave the clinics. Shah et al. (2012) used an SVM for a more complex problem than just classification by doing voxel based analyses for the localization of PCa. The drawback of this model is that for training, a large amount of imaging data must be available, as well as 3D pathology, but the advantage is that the usage of subregions in the prostate allows for data augmentation.

Notable is that a number of nomograms initially developed more than two decades ago are currently still in use, though updated using newer datasets. The continued application of simple, easy to interpret models is something to keep in mind in the development of new mDSS. Though artificial intelligence has the potential to improve diagnosis of PCa, transparency plays a large role in clinical application. It also shows that predictive parameters for PCa are very consistent, with persistent usage of Gleason score, PSA, clinical stage, and age. Any new mDSS being developed should be tested against the performance of these parameters to avoid tackling a simple problem using computationally heavy machine learning.

When looking at the available treatment mDSS, we see that most of these are focused on RT. This is likely due to the patient specific treatment planning done in RT, which results in highly detailed dose maps before the start of treatment. This is ideal for mDSS, as different treatment plans can be directly compared, and the different outcomes can be predicted using dose response curves. Other types of treatment, such as prostatectomy or watchful waiting, rely much more heavily on clinical parameters and tumor parameters for predictions of their outcome or on subjective physician decisions. This makes it harder to compare outcomes of different treatments for the same patient. The development of new mDSS comparing completely different treatment types with each other for the same patient would be very beneficial for filling this gap in the current literature.

### 4.3. Study Limitations

This study focused on mDSS in PCa, and the scope did not include any other cancer types. It is possible that general mDSS, applicable for more than one cancer type, were therefore overlooked. The search was also focused on mDSS and thus did not include any predictive models that could aid in decision-making. The terms describing mDSS may not have been used in interesting studies that could have been included in this overview. ‘Patient decision aids' was also not a search term, which may be the cause of the limited number of mDSS for patients included in this overview. The search performed was only MEDLINE/PubMed linked, so studies not available on these media were overlooked.

Inherent to literature overviews is that negative findings are not always reported, so failed attempts at creating mDSS are often uncommon in overviews and literature reviews, and this may cause a biased view on the subject.

We assessed the reporting quality of each included study using the TRIPOD statement for quantification and comparison. Although the TRIPOD statement has been endorsed by a large number of medical journals and editorial organizations, it is not a universal gold standard. The checklist used for the assessment has limitations, such as the severe point punishment for the lack of specific keywords in title or abstract. This could reduce the score of a well-written paper. Additionally, the adherence to the TRIPOD statement was compressed into a single number, and the specific reporting issues were not named, such as improper analyses, lack of validation, or reporting of study specifics. However, it is a useful tool to show the strength of a report, as when adherence to the TRIPOD statement was high, the paper was clearly written, and all proper steps were taken for the development of quality mDSS tools.

## 5. Conclusion

A number of mDSS for the primary diagnosis and staging of localized PCa are available. Treatment mDSS were mostly focused on EBRT, for which several tools are available. However, a lack of mDSS for other treatment modalities suggests that the development of new tools is necessary to objectively compare different treatment modalities. The development of patient decision aids is a new field of research, and few successes have been made for PCa patients. Though the idea of informed decision-making by patients is in line with the goal of personalized medicine, the development of these tools needs to overcome a number of barriers to be successful, like comprehensiveness, language barriers, patient cooperation, and physician cooperation. More research needs to be performed to better empower clinical decisions in the diagnosis and treatment process.

## Figures and Tables

**Table 1:** Overview of diagnosis support systems.

Study	N	Decision/Diagnose	Inputs	Outcomes	TRIPOD
(Roach et al. 1994)[25]	282	Low or high risk of LN involvement	PSA,GS, Clinical stage	P LN involvement	79%

(Diaz et al. 1994)[26]	217	Low or high risk of SV involvement	PSA, GS	P SV involvement	69%

(D'Amico et al. 1998)[27]	1872	Patient risk group	PSA, GS, Clinical stage	5-year PSA outcome	72%

(Chang et al. 1999)[28]	43	Localized vs Advanced PCa	PSA, GS, TRUS, DRE	P advanced PCa and P localized PCa	65%

(Roach et al. 2000)[29]	895	Extracapsular extension	PSA, GS	Extracapsular extension risk	92%^*∗∗*^

(Lee et al. 2010)[30]	1077	Biopsy	Clinical^*∗*^, Imaging^*∗*^, PSA	P PCa	86%

(Kim et al. 2011)[31]	532	Advanced PCa	TRUS, Clinical, PSA	P advanced PCa	79%

(Shah et al. 2012)[32]	31	Location PCa	MRI image	Cancer probability map	83%

(Mukai et al. 2013)[33]	30773	PSA test	Clinical	Recommendation	-^*∗∗∗*^

(Sadoughi et al. 2014)[34]	360	PCa	PSA, Age	P PCa	33%

(van Leeuwen et al. 2017)[35]	591	Significant PCa	Clinical, PSA, PIRADS, DRE	P significant PCa	97%

(Tosoian, et al. 2017)[36]	4459	Pathological Stage	PSA, GS, Clinical stage	% likelihood of given stage	83%

Abbreviations. N: number of patients; P: probability; PCa: prostate cancer; PSA: prostate specific antigen; PIRADS: Prostate Imaging Reporting and Data System; TRUS: transrectal ultrasound scan; LN: lymph node; GS: Gleason score; SV: seminal vesicles; TRIPOD: adherence to the TRIPOD statement; DRE: digital rectal examination.

^*∗*^Clinical, imaging, and tumor parameters.

^*∗∗*^Abstract only.

^*∗∗∗*^No development or validation of mDSS: no TRIPOD evaluation possible.

**Table 2:** Overview of treatment support systems.

Study	N	Decision	Inputs	Outcomes	TRIPOD
(Hodges et al. 2012)[43]	Model	SBRT, IMRT	Utility, transitions	QALY, Costs, ICER	80%

(Reed et al. 2014)[44]	Model	ART	Risk group ProsVue slope	QALY, Cost, ICER	80%

(Vanneste et al. 2015)[45]	Model	IMRT+IRS, IMRT	Utility, transitions	QALY, Cost, ICER	81%

(Smith et al. 2016)[46]	Model	RT plan	DVH, Clinical^*∗*^	TCP, NTCP, QALY	87%

(van Wijk et al. 2018)[47]	23	IRS in RT	DVH, Clinical	TCP, NTCP	84%

(Salem et al. 2018)[48]	200	Follow-up	Symptoms, Blood tests	Follow-up suggestion	71%

(Walsh et al. 2018)[49]	25	IMRT, V-mat, PSPT, IMPT	DVH	TCP, NTCP, Robustness, stability	84%

Abbreviations. N: number of patients; IMRT: intensity modulated radiotherapy; V-mat: volumetric-modulated arc therapy; PSPT: passively scattered proton therapy; IMPT: image modulated proton therapy; TCP: tumor control probability; NTCP: normal tissue complication probability; DVH: dose-volume histogram; QALY: quality adjusted life year; IRS: implantable rectum spacer; ART: adjuvant radiotherapy; ICER: incremental cost-effectiveness ratio; TRIPOD: adherence to the TRIPOD statement.

^*∗*^Clinical parameters.

**Table 3:** Summary of patient support systems.

Study	N	Decision	Inputs	Outcomes	TRIPOD
(Nguyen et al. 2009)[50]	Literature	Treatment	Various	Various	86%

(Berry et al. 2013)[51]	494	Treatment	P3P	Decision satisfaction	82%

Abbreviations. N: number of patients; P3P: personal patient-profile for prostate cancer.

**Table 4:** Overview of excluded studies that described tools to improve mDSS.

Study	N	Tool	Inputs	Outcomes
(Daemen et al. 2009)[61]	55	Genetic integration	DNA, CNV	Cancer outcome

(Kuru et al. 2013)[59]	50	Diagnostics	mpMRI	PIRADS

(Zumsteg et al. 2013)[55]	1024	Risk stratification	Risk factors, Gleason score, biopsy	Risk group

(Beyan et al. 2014) [62]	Model	Genetic integration	SNPs	Various

(Kent and Vickers 2015)[60]	Model	Diagnostics	Clinical and tumor features	Life expectancy

(Gnanapragasam et al. 2016)[56]	10139	Risk stratification	PSA, stage, Gleason score	Risk group

(Epstein et al. 2016)[57]	26325	Risk stratification	PSA, stage	Gleason grade

(Gries et al. 2017)[58]	120	Utility values	Utility's 18 health states	Utility's 243 heath states

Abbreviations. N: Number of patients; CNV: copy number variation; SNP: single nucleotide polymorphism; PSA: prostate specific antigen; mpMRI: multiparametric magnetic resonance imaging; PIRADS: Prostate Imaging Reporting and Data system.

## Supplementary Materials

Supplementary Materials The supplementary material we provided contains a checklist of TRIPOD points and the calculation method we used to calculate the adherence to the TRIPOD statement. The version put in the supplementary materials is the one available on the TRIPOD site. It first contains a description, then part A with general description of the study, and then part B where each point is checked.
